# Expression characteristics and potential function of non-coding RNA in mouse cortical cells

**DOI:** 10.3389/fnmol.2024.1365978

**Published:** 2024-04-10

**Authors:** Yanrong Wei, Junjie Lei, Yujie Peng, Huizhong Chang, Ting Luo, Yuanchun Tang, Lifang Wang, Huiying Wen, Giacomo Volpe, Longqi Liu, Lei Han

**Affiliations:** ^1^College of Life Sciences, University of Chinese Academy of Sciences, Beijing, China; ^2^BGI Research, Hangzhou, China; ^3^BGI College & Henan Institute of Medical and Pharmaceutical Sciences, Zhengzhou University, Zhengzhou, China; ^4^School of Biology and Biological Engineering, South China University of Technology, Guangzhou, China; ^5^Hematology and Cell Therapy Unit, IRCCS–Istituto Tumori ‘Giovanni Paolo II’, Bari, Italy; ^6^BGI Research, Shenzhen, China

**Keywords:** non-coding RNA, single-cell SMART-Seq v4, cerebral cortex, cell-type specific noncoding RNA, spatial transcriptome, hdWGCNA, neurological disorders

## Abstract

Non-coding RNAs (ncRNAs) play essential regulatory functions in various physiological and pathological processes in the brain. To systematically characterize the ncRNA profile in cortical cells, we downloaded single-cell SMART-Seq v4 data of mouse cerebral cortex. Our results revealed that the ncRNAs alone are sufficient to define the identity of most cortical cell types. We identified 1,600 ncRNAs that exhibited cell type specificity, even yielding to distinguish microglia from perivascular macrophages with ncRNA. Moreover, we characterized cortical layer and region specific ncRNAs, in line with the results by spatial transcriptome (ST) data. By constructing a co-expression network of ncRNAs and protein-coding genes, we predicted the function of ncRNAs. By integrating with genome-wide association studies data, we established associations between cell type-specific ncRNAs and traits related to neurological disorders. Collectively, our study identified differentially expressed ncRNAs at multiple levels and provided the valuable resource to explore the functions and dysfunctions of ncRNAs in cortical cells.

## Introduction

Non-coding RNAs (ncRNAs) comprise a substantial part of genome transcription, but do not possess the capacity to encode proteins (Yang et al., 2012). Although some ncRNAs might exhibit structural similarities to genes, those are classified as pseudogenes (Tay et al., 2014). ncRNAs are generally subdivided into small non-coding RNA (sncRNA) and long non-coding RNA (lncRNA) according to their length, the first being around 21–25 nucleotide and the latter usually longer that 200 nucleotides (Losko et al., 2016; Bhat et al., 2020). ncRNAs are abundantly expressed in several cell types of the mammalian brain (Derrien et al., 2012; Ransohoff et al., 2018; de Goede et al., 2021), shaping a distinctive and dynamic molecular profile of the brain through diverse regulatory mechanisms (Zimmer-Bensch, 2019; Srinivas et al., 2023). Previous inspection of ncRNAs roles has mainly relied on *in situ* hybridization (ISH) (Mercer et al., 2008) and bulk RNA-sequencing (RNA-seq) technologies (Kadakkuzha et al., 2015; Isakova et al., 2020), which are not adequate enough for comprehensively examining ncRNAs specificity and their involvement in intricate regulatory mechanisms governing cerebral cortex’s function. To date, a comprehensive compendium of cell-specific roles of ncRNAs in the adult mouse cerebral cortex is currently lacking.

In recent years, the continuous development of single-cell technologies has significantly advanced our understanding of ncRNA at the single-cell level (Petropoulos et al., 2016; Hwang et al., 2018). In comparison to other 3’ RNA sequencing methods (Ziegenhain et al., 2017; Zhang et al., 2019), SMART-Seq v4 utilizes full-length coverage across transcripts thus enabling the capture of a greater number of genes, including ncRNAs (Ziegenhain et al., 2017; Song et al., 2018; Wang et al., 2021). Such technology facilitates the identification of previously uncharacterized ncRNAs specific to particular cell types. Accumulating evidence has demonstrated that a limited set of ncRNAs display cell type and layer-specific expression patterns in the cerebral cortex (Liu et al., 2016). For instance, *DLX6-AS1* has been observed to be specifically expressed in interneurons, while *AK017893* and *AK159011* were found to be abundant in layer 2/3 and layer 5, respectively (Mercer et al., 2008; Liu et al., 2016). In addition, the existence of regional specificity of ncRNA within the cortex remains to be elucidated.

Whilst ncRNAs are involved in a wide range of biological processes in the brain (Kleaveland et al., 2018; Nie et al., 2019; Mehta et al., 2020; Li et al., 2022; Wu et al., 2022), their specific functions remain largely elusive compared to protein-coding genes (pcGs). Genome-wide association studies (GWAS) have identified numerous genetic variants that are associated with neuropsychiatric disorders, most of which are located in noncoding regions of the genome (Han et al., 2022; Morris et al., 2023). Previous study has reported that individuals with autism spectrum disorder (ASD) carrying the rs4307059 T allele exhibit increased expression of *MSNP1AS*, suggesting that high levels of the *MSNP1AS* transcript might contribute to the risk of ASD. These variants may disrupt critical neuronal processes and contribute to the pathogenesis of neuropsychiatric diseases (Kyzar et al., 2022). Therefore, it is paramount to further investigate the association between cell type-specific ncRNAs and mental disorders in order to comprehensively understand the role of ncRNAs in the development of neurological diseases.

In the present study, we have generated a comprehensive map of ncRNA specificity at multiple levels, including cell types, cortical layers and cortical regions in the adult mouse brain using the SMART-Seq v4 data. By constructing a gene co-expression network and performing gene ontology (GO) functional enrichment analysis, we have predicted the potential biological processes in which ncRNAs may participate. Additionally, we have uncovered the potential connection between neurological diseases and cell type specific ncRNAs. Our findings provide valuable insights for both basic and clinical research on ncRNAs.

## Materials and methods

### Data acquire, quality control and clustering

The single-cell RNA-seq expression matrix and meta file of adult mouse cortex (~8 week-old male and female mouse) was downloaded from the website.^1^ According to the provided brain region annotation, we first extracted data from 18 cortical regions, including ACA, AI, AUD, CLA, ENTl, ENTm, GU, MOp, MOs-FRP, ORB, PL-ILA, PTLp, RSP, SSp, SSs, TEa-PERI-ECT, VIS, and VISp. According to the metadata annotation, we removed outlier cells and cell types with a cell count less than 50. In order to facilitate subsequent analysis, we merged cells from different brain regions but belonging to the same cell type, including L2/3 IT (L2 IT ENTl, L2 IT RHP, L2/3 IT ENTl, L2/3 IT CTX-1, L2/3 IT CTX-2 and L3 IT ENT), L5 IT (L5 IT TPE-ENT and L5 IT CTX), L6b/CT (L6b CTX and L6b/CT ENT), and Sst (Sst and Sst Chodl) and finally we obtained 24 cell types.

The single-cell expression data matrix (total RNA matrix) was then quality controlled. Filtered total RNA matrix was normalized using the calculateTPM functions from the scuttle package (v1.8.0) (McCarthy et al., 2017). According to the genome annotation file, the single-cell expression data matrix was divided into a pcG matrix and a ncRNA matrix. The ncRNA gene type includes three categories, namely lncRNA, sncRNA (including misc_RNA, scaRNA, snRNA, miRNA and snoRNA) and pseudogene (pseudogene, transcribed_unitary_pseudogene, unitary_pseudogene, translated_unprocessed_pseudogene, unprocessed_pseudogene, processed_pseudogene, transcribed_processed_pseudogene, and transcribed_unprocessed_pseudogene).

Global clustering of the mouse cortex dataset was performed using Seurat package (v4.3.0) (Hao et al., 2021) in a R environment (v4.2.2). Filtered data were normalized using the calculateTPM functions form the scuttle package (v1.8.0) (McCarthy et al., 2017), and the highly variable genes were selected according to their average expression and dispersion. Each gene was scaled with default options and Principal Component Analysis (PCA) was used to linear dimensionality reduction. UMAP using 30 principal components was used for non-linear dimensionality reduction to visualize the data. Lastly, clustering was performed to determine the optimal resolution for clustering the pcG (res = 2.0) and ncRNA (res = 2.0) separately, based on the resolution that yielded the clearest clusters.

### Acquisition of 10x V2 (left) / V3 (right) single-cell/singe-nucleus RNA sequencing data

We obtained gene expression of adult mouse brain MOp single-cell/singe-nucleus transcriptome data from https://nemoanalytics.org/index.html?multigene_plots=0&gene_symbol_exact_match=1&gene_symbol=Aldh1a3.

### Acquisition and processing of spatial transcriptome data

The adult mouse brain (Sagittal) spatial transcriptome data with the Visium platform can be acquired from https://www.10xgenomics.com/datasets?menu%5Bproducts.name%5D=Spatial%20Gene%20Expression&query=&page=1&configure%5BhitsPerPage%5D=50&configure%5BmaxValuesPerFacet%5D=1000. The data was processed according to the standard process provided by Seurat (v4.3.0) (Hao et al., 2021), including quality control, data filtering, dimensionality reduction, and clustering using BayesSpace (v1.6.0) (Zhao et al., 2021). Then, Data were annotated based on expression of known layer marker genes.

### Differential expression analysis

Analysis of differentially expressed genes was performed with the FindAllMarkers function in the Seurat package (v4.3.0) (Hao et al., 2021) to examine differences across different cell types, cortical layers, and cortical areas. The Benjamini and Hochberg (BH) algorithm was used to correct the false discovery rate (FDR) during the analysis. Differentially expressed genes were defined as genes with a log2FC > 0.25 and FDR < 0.05.

### High dimensional weighted gene co-expression network analysis

High dimensional weighted gene co-expression network analysis (hdWGCNA) was used to construct a scale-free network at single cell level by R package ‘hdWGCNA’ (v0.2.1) (Morabito et al., 2023). First, metacells were constructed by the k-Nearest Neighbors (KNN) algorithm. Then, the data was normalized using the calculateTPM functions from the scuttle package. Gene modules were identified by Construct Network with soft_power = 5. Hub genes were identified as the most connected genes within each module. Module scores were assigned using the ModuleExprScore function based on the genes contained in each module. Finally, an interaction network was constructed by extracting all ncRNAs in each module and their top 10 most highly correlated genes. The Cytoscape software (v3.9.1) was used for visualization of this network.

### Functional annotation gene ontology term analysis

GO enrichment analysis for biological process (BP) was performed using the clusterProfiler software package (v4.2.2) on identified gene modules. GO terms with a false discovery rate (FDR) < 0.05 were considered to be significantly enriched.

### Association of human GWAS and genetic disease data with mouse cortical cell types

In order to test the enrichment of genes related to human neurological disorders and traits for each cell type, we performed linkage disequilibrium (LD) score regression analysis as previously described.^2^ Then, we considered DE pcGs and DE ncRNAs with an adjusted FDR < 0.05 and log2FC > 0.1 in each cell type and converted the genome coordinates of GRCm38 into hg19 genome coordinates by this website.^3^ The summary statistics file for each trait was downloaded from the UK Biobank database or published studies (Supplementary Table S7). To calculate cell-type-specific LD scores, we first created annotation files for 22 chromosomes in each cell type with script make_annot.py using options --bed-file --bimfile 1000G.EUR.QC.bim --annot-file. Then, the annotation files were used as input to compute LD scores with the ldsc.py script using options --l2 --bfile 1000G.EUR.QC --ld-wind-cm 1 --annot --thin-annot --print-snps. Next, we ran the ldsc.py script with the --h2-cts flag to perform regressions following the standard workflow.^4^ We report the coefficient *p* value as a measure of the association of each cell type with the traits. All plots show the −log-transformed *p*-value of partitioned LD score regression.

## Results

### Profiling of non-coding RNA in mouse cortical cells

**Figure 1:**
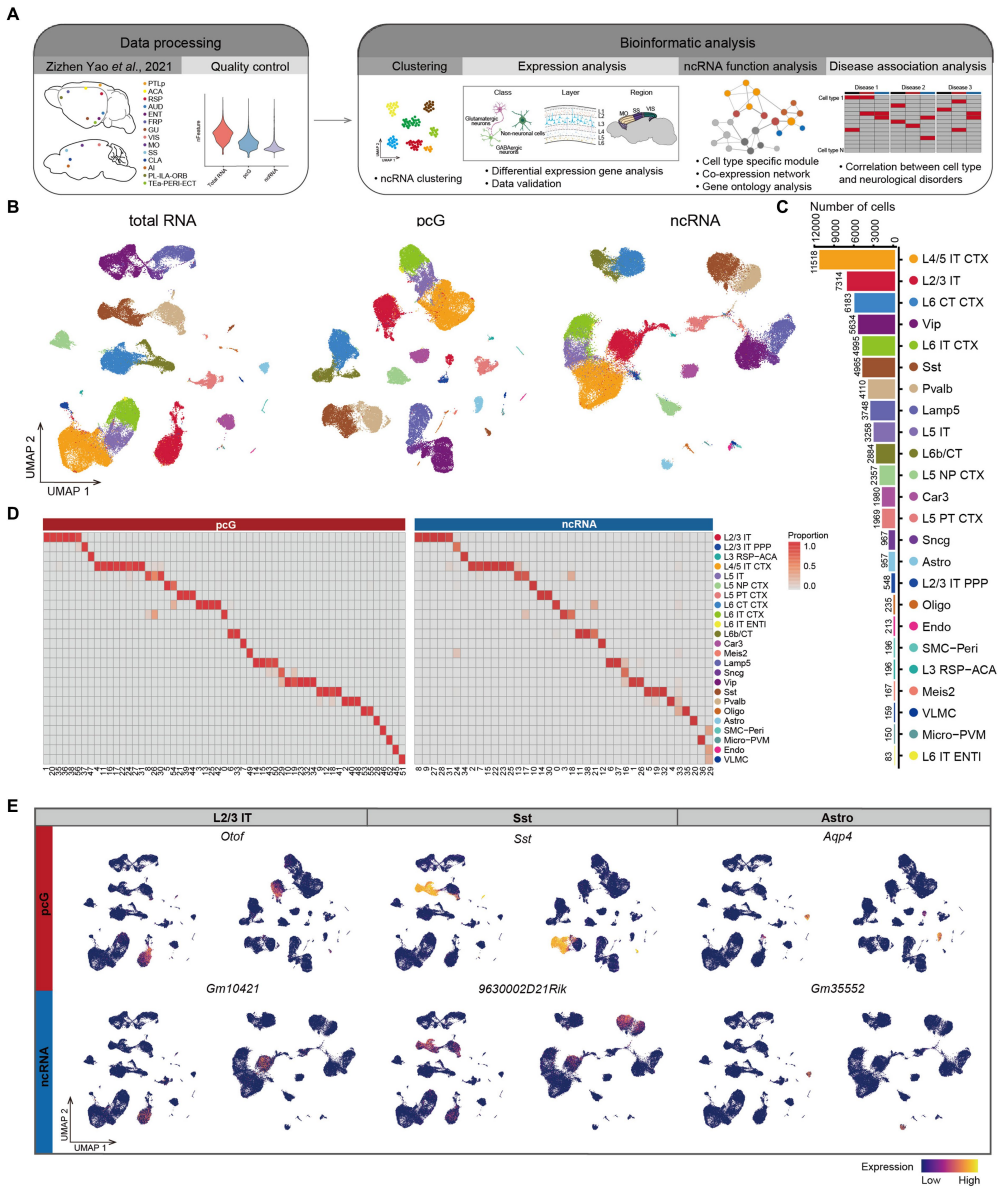
Expression specificity of ncRNA across mouse cerebral cortex. **(A)** Schematic overview of the analysis workflow for SMART-Seq v4 dataset in mouse cerebral cortex. **(B)** UMAP visualization of all cells clustered using total RNA (left), protein coding gene (pcG) (middle) and noncoding RNA (ncRNA) (right), colored by 24 cell types. Bottom: Histogram showing the number of cells for each cell type. L2/3 IT, Layer 2/3 intratelencephalic neuron; L2/3 IT PPP, Layer 2/3 intratelencephalic neuron in postsubiculum, presubiculum and parasubiculum areas; L3 RSP-ACA, Layer 3 glutamatergic neuron in retrosplenial and anterior cingulate areas; L4/5 IT CTX, Layer 4/5 intratelencephalic neuron in isocortex; L5 IT, Layer 5 intratelencephalic neuron; L5 NP CTX, Layer 5 near-projecting neuron in isocortex; L5 PT CTX, Layer 5 pyramidal tract neuron in isocortex; L6 CT CTX, Layer 6 corticothalamic neuron in isocortex; L6 IT CTX, Layer 6 intratelencephalic neuron in isocortex; L6 IT ENTL, Layer 6 intratelencephalic neuron in lateral entorhinal area; L6b/CT, Layer 6b/corticothalamic neuron; Car3, Car3 glutamatergic neuron; Meis2, Meis2 GABAergic neuron; Lamp5, Lamp5 GABAergic neuron; Sncg, Sncg GABAergic neuron; Vip, Vip GABAergic neuron; Sst, Sst GABAergic neuron; Pvalb, Pvalb GABAergic neuron; Oligo, Oligodendrocyte; Astro, Astrocyte; SMC-Peri, Smooth muscle cell-Pericyte; Micro-PVM, Microglia-perivascular macrophage; Endo, Endothelial cell; VLMC, Vascular and leptomeningeal cell. **(C)** Histogram showing the number of cells for each cell type. **(D)** The heatmap showing the percentage of cell types in the cluster clustered by pcG (left) and ncRNA (right). **(E)** UMAP visualization of pcGs (top) and ncRNAs (bottom) specifically expressed in L2/3 IT, Sst and Astro.

The mammalian cerebral cortex is composed of diverse cell types that are characterized by distinct molecular profiles (di Bella et al., 2021). In order to investigate the expression patterns of ncRNAs in different cell types within the mouse cerebral cortex, we analyzed publicly available SMART-Seq v4 single cell RNA-seq (scRNA-seq) data (Tasic et al., 2018; Yao et al., 2021b) from 18 cortical regions of approximately 8 week-old mice (Figure 1A). Those include the anterior cingulate area (ACA), agranular insular cortex (AI), auditory cortex (AUD), claustrum (CLA), lateral entorhinal cortex (ENTl), medial entorhinal cortex (ENTm), gustatory cortex (GU), primary motor cortex (MOp), secondary motor cortex and frontal pole cortex (MOs-FRP), orbital cortex (ORB), prelimbic and infralimbic cortex (PL-ILA), posterior parietal association cortex (PTLp), retrosplenial cortex (RSP), primary somatosensory cortex (SSp), supplemental somatosensory cortex (SSs), temporal association-perirhinal-ectorhinal cortex (TEa-PERI-ECT), visual cortex (VIS), and primary visual cortex (VISp). In this dataset, a total of 31,785 genes were detected, with pcGs accounting for the majority with 20,189 genes (63.5%). ncRNAs accounted for 11,596 genes (36.5%), which were further classified into three major categories, those being 3,865 lncRNAs (12.2%), 1,025 sncRNAs (3.2%), and 6,706 pseudogenes (21.1%) (Supplementary Figure S1A).

The profiling of mouse cerebral cortex by using total RNA (Yao et al., 2021b), was conducted on a total of 71,234 individual cells which were clustered in 24 cell types (Figure 1B), including 12 types of glutamatergic excitatory neurons (Glu), 6 types of GABAergic inhibitory neurons (GABA), and 6 types of non-neuronal cells (Non-neu), covering most of cell types within the cortex. The number of cells for each of these 24 cell types ranged from 11,518 for L4/5 IT CTX to 83 for L6 IT ENTL (Figure 1C). We sought to assess whether the expression of ncRNAs alone would lead to the same cell type identification. To do so, we first normalized the total RNA matrix and divided the data into two matrices based on gene type into a pcG matrix and ncRNA matrix. Both were then subjected to clustering, resulting in the identification of 57 clusters for pcG matrix and 39 clusters for ncRNA matrix (See methods). We then projected the cell identities obtained by total RNA profiling onto each cell cluster identified by uniform manifold approximation and projection (UMAP) of pcG and ncRNA, respectively (Figure 1B). We observed that the average number of genes detected per cell were higher for pcGs (gene, 8,232) than that of ncRNAs (gene, 1,024) (Supplementary Figure S1B). From a cell type perspective, we noticed that the number of both pcGs and ncRNAs detected in neuron was generally higher compared to Non-neu (Supplementary Figure S1C). To examine correlation of cell clusters identified by either pcGs or ncRNAs and cell types defined by total RNA profiling, we calculated the proportion of each cell type in different clusters and found that clusters with a high proportion of the same cell type tended to cluster together (Figures 1B,D and Supplementary Figure S1D), suggesting that the expression of ncRNAs alone is sufficient to distinguish most cell types and that this capacity is roughly equivalent to that of pcGs, despite the number of ncRNAs detected in each cell type being much lower than pcGs.

Next, we performed differential expression analysis separately for pcG and ncRNA in each cell type and detected a set of cell type-specific pcGs and ncRNAs (Supplementary Figure S1E), such as *Otof* (pcG) and *Gm10421* (ncRNA) in L2/3 IT, *Sst* (pcG) and *9630002D21Rik* (ncRNA) for Sst neuron, and *Aqp4* (pcG) and *Gm3555*2 (ncRNA) for Astro (Figure 1E). Many of these pcGs have been previously reported as marker genes specific to particular cell type (Kozareva et al., 2021; Zhang et al., 2021; Yao et al., 2021a), providing support for the cell type specificity of ncRNAs.

### Cell class/cell type specific ncRNA in mouse cerebral cortex

**Figure 2:**
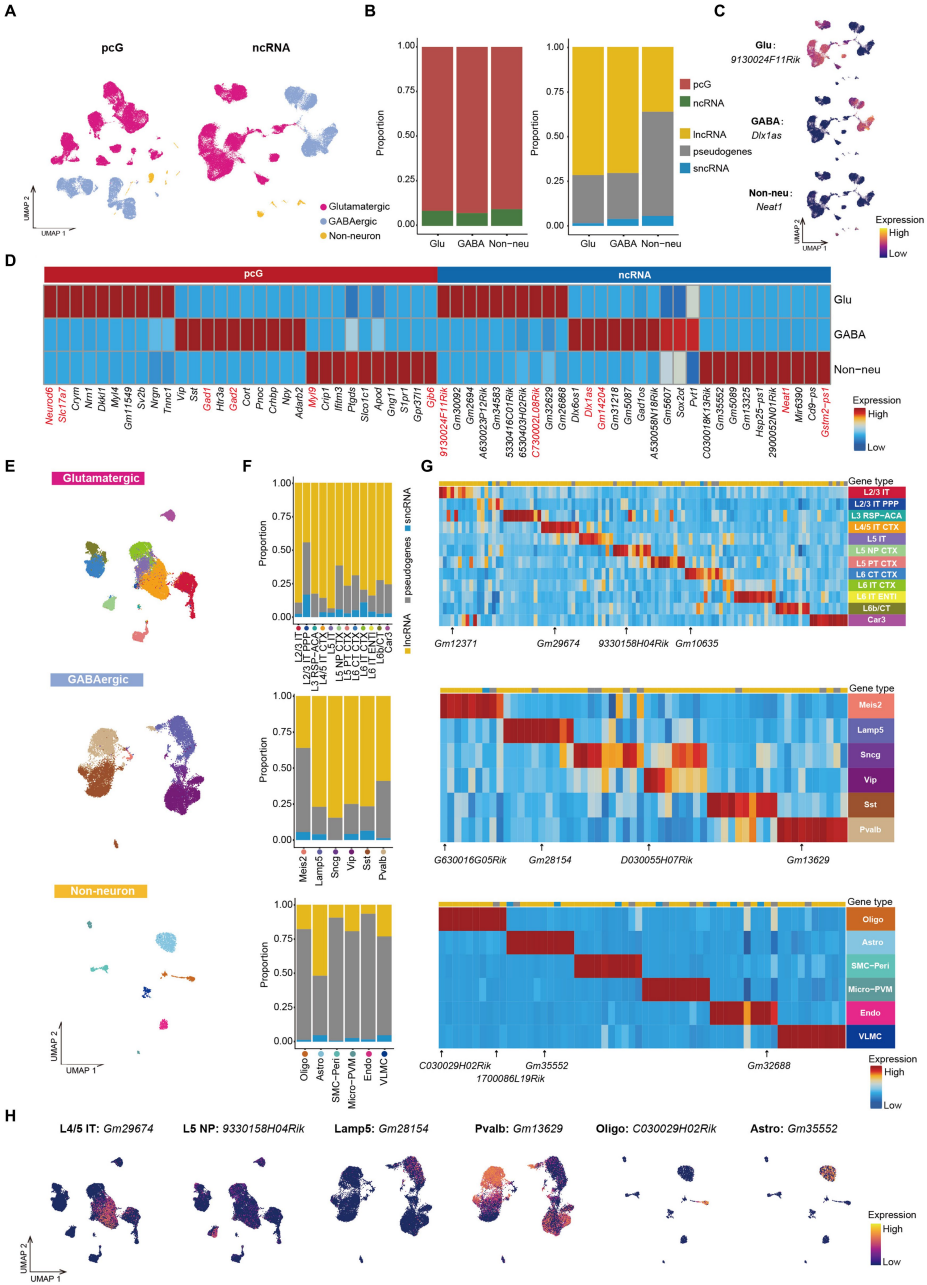
ncRNAs specifically expressed in Glu/GABA/Non-neu. **(A)** UMAP visualization of Glu, GABA and Non-neu using pcG (left) and ncRNA (right). **(B)** Stacked bar plots showing the proportion of differentially expressed (DE) gene type in Glu/GABA/Non-neu. Left, pcG and ncRNA. Right, ncRNA type including lncRNA, sncRNA and pseudogene. **(C)** UMAP visualization of ncRNAs specifically expressed in Glu, GABA, and Non-neu. **(D)** Heatmap showing the top DE pcGs and ncRNAs of each class. Known marker pcGs and ncRNAs shown in panel **(C)** and Supplementary Figure S2C are marked in red. **(E)** UMAP visualization of all cells clustered using ncRNA, colored by Glu cell type (top), GABA cell type (middle) and Non-neu cell type (bottom). **(F)** Stacked bar plots showing the proportion of DE ncRNA type in Glu cell type (top), GABA cell type (middle) and Non-neu cell type (bottom). **(G)** Heatmap showing the top DE ncRNA in Glu cell type (top), GABA cell type (middle) and Non-neu cell type (bottom). ncRNAs shown in panel **(H)** and Supplementary Figure S2I are marked. **(H)** UMAP visualization of ncRNAs specifically expressed in L4/5 IT, L5 NP, Lamp5, Pvalb, Oligo, and Astro.

The mouse cerebral cortex consists mainly neurons and Non-neu, with neurons further categorized as Glu and GABA (Delgado et al., 2022; Wei et al., 2022). The majority of cortical cells profiled in this study are neurons (Glu, cell number: 43,285, cell percentage: 67%; GABA, cell number: 19,591, cell percentage: 30%), while only 1,910 cortical cells were identified as Non-neu, accounting for roughly 3% (Supplementary Figure S2A). This profound unbalance in the percentage of the cells captured can be attributed to the use of fluorescence activated cell sorting (FACS) which selectively enriches for neurons. To perform a profiling based on ncRNA expression in different population of cortical cells, we collapsed the 24 cell types identified into three major classes of cortical cells, that is Glu, GABA and Non-neu, and projected them on UMAP clustering those by either pcG or ncRNA expression (Figure 2A). Next, we performed differential expression analysis among these 3 classes of cells (Supplementary Table S1) to identify cell type-specific pcGs or ncRNAs. Notably, we observed that Glu displayed the highest cell number of differentially expressed (DE) pcGs and ncRNAs (Supplementary Figure S2B), possibly owing this to more number of cell types in Glu. The ratio of DE pcGs was higher than that of ncRNAs in all three cell classes (Figure 2B). Additionally, the number of DE lncRNAs and pseudogenes was much higher than that of sncRNAs in all three cell classes (Figure 2B), this difference possibly being attributed to the inability of technology to adequately capture shorter ncRNAs. Several cell type-specific pcGs are well-known (Zhang et al., 2021), such as *Neurod6* and *Slc17a7* in Glu, *Gad1* and *Gad2* in GABA, *Myl9* and *Gjb6* in Non-neu (Figure 2D). Similarly, we also identified several ncRNAs specifically expressed in distinct cell types. For instance, we detected *9130024F11Rik* and *C730002L08Rik* expression in Glu, *Dlx1as*, *Gm14204* and *Pvt1* in GABA while *Neat1* and *Gstm2-ps1* were uniquely detected in Non-neu (Figures 2C,D and Supplementary Figure S2C). Our observations are in agreement with previous studies in which *Gm14204* and *Dlx1as* were reported to be specifically expressed in GABA (Fukumoto et al., 2018; Li et al., 2018). *Dlx1as*, as an antisense ncRNA of *Dlx1*, plays an important role in regulating the transcriptional level and stability of *Dlx1*, a transcription factor that determines the fate of GABAergic neurons (Kraus et al., 2013). ncRNA *Pvt1* is highly expressed in GABA, and it has been confirmed to have a regulatory effect on human neuronal differentiation (Wu et al., 2022, 2023), indicating its role in the lineage commitment of GABA neurons.

Having observed that ncRNAs are sufficient to discriminate specific classes of cells, we next surveyed whether ncRNAs can be used to define specific cell type identities by re-clustering cells from each cell class on the basis of ncRNA expression only. We projected the cell type annotation obtained by total RNA profiling to the re-clustered cells and observed that cells with same annotation tended to accumulate together to form distinct clusters (Figure 2E), indicating that both neuronal and non-neuronal cell types can be effectively distinguished by the expression of ncRNA. Furthermore, we noticed that ncRNAs are not only able distinguish these cell types but also provide a way to further divide those cell type into different subclusters (Figure 2E). For example, Micro-PVM could be clearly divided into two populations (Supplementary Figure S2D), that, based on the use of canonical marker genes (Yang et al., 2019; Prinz et al., 2021; Jeong et al., 2022; Supplementary Figure S2E), can be annotated as Micro and PVM. Those cell type could also be distinguished based on the distinct expression of *Gm33858* (Micro) and *Gm1966* (PVM) ncRNAs (Supplementary Figure S2F). We then performed ncRNAs differential expression analysis in different cell types and calculated the proportion of each gene type. We found that the ratio of pseudogenes was much less in cortical IT neurons except for L2/3 IT PPP (Figure 2F). In GABA cells, we observed that the ratio of pseudogenes in Meis2 and Pvalb was higher compared to other cell types (Figure 2F). We also observed that the proportion of pseudogenes in non-neuronal cell types was higher than 75% with the exception of Astro in which those accounted for roughly 50% (Figure 2F). Instead, sncRNAs displayed the lowest ratio in all cell types (Figure 2F).

Additionally, we also screened DE ncRNAs in each cell type (Figure 2G) identifying, for example, the specific expression of *Gm12371* in L2/3 IT, *Gm29674* in L4/5 IT CTX, *9330158H4Rik* in L5 NP, *Gm10635* in L6 CT CTX, *Gm2815*4 in Lamp5, *Gm13629* in Pvalb, *D030055H07Rik* in Vip, *1700047M11Rik* and *C030029H02Rik* in Oligo, *Gm35552* in Astro, and *Gm32688* in Endo (Figure 2H and Supplementary Figure S2I). These cell type-specific ncRNAs could also be confirmed in other independent scRNA-seq and snRNA-seq data (Supplementary Figure S2G) from the adult mouse MOp (Supplementary Figures S2G–I; Yao et al., 2021a). Taken together, we identified 1,600 cell type specific ncRNAs (Supplementary Table S2) and provided a resource for further exploring the function of ncRNAs in different cell types.

### Layer specific ncRNA in mouse cerebral cortex

**Figure 3:**
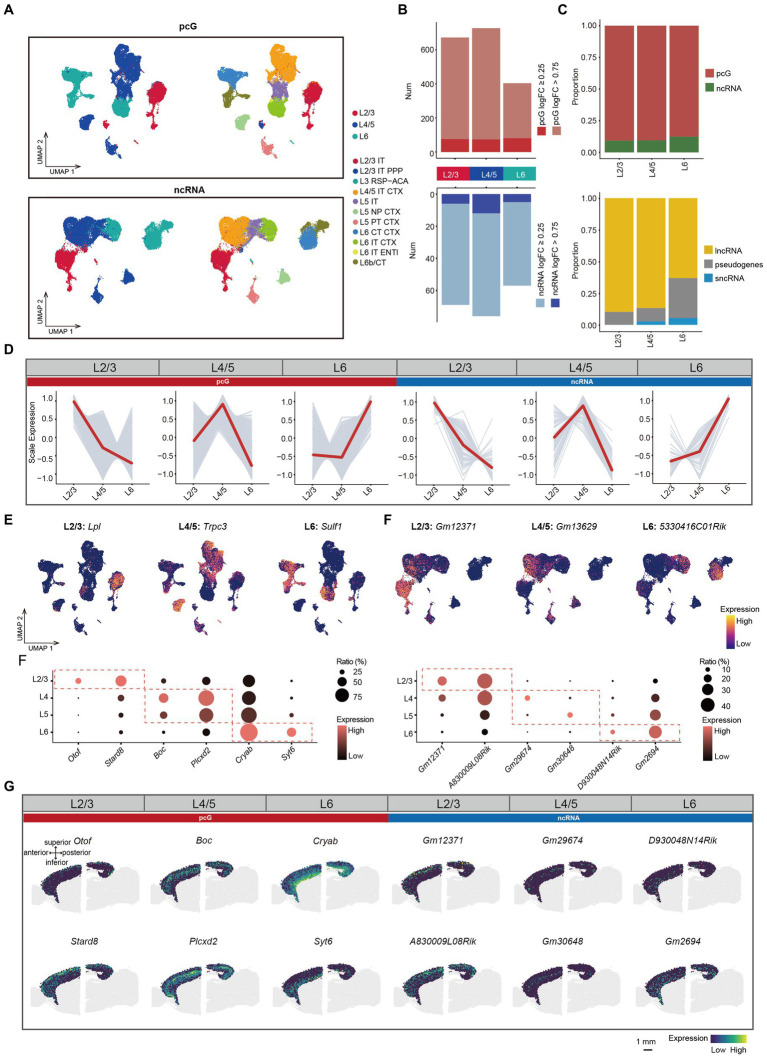
ncRNAs specifically expressed in different layers. **(A)** UMAP visualization of all cells clustered using pcG (top) and ncRNA (bottom), colored by layer (left) and cell type (right). **(B)** Histogram showing the number of DEGs per layer with pcG (red) and ncRNA (blue). DE genes were defined as genes with log2(fold change) > 0.25 (light color bars) or > 0.75 (dark color bars) and FDR-adjusted *p*-value <0.05. **(C)** Stacked bar plots showing the proportion of DE gene type in different Layers. Top, pcG and ncRNA. Bottom, ncRNA type including lncRNA, sncRNA and pseudogenes. **(D)** Line plot showing layer-specifically expressed pcGs and ncRNAs. Gray lines represent the expression dynamics of individual genes and the red line represents the average expression in different layers. **(E)** UMAP visualization of DE pcGs and ncRNAs specifically expressed in Layer (L) 2/3, L4/5 and L6. **(F)** Bubble plot showing layer-specific expression of pcGs (left) and ncRNAs (right) in different layers of 10x Visium ST data from adult mouse brain. The color of each bubble indicates the average expression level, and the size indicates the proportion of expressing cells. **(G)** Spatial visualization of the layer-specific pcGs and ncRNAs shown in F expressed in adult mouse cerebral cortex of 10x Visium ST data. Scale bar, 1 mm.

In the mouse cerebral cortex, the Glu represent the largest neuronal population and exhibit a distinct laminar preference across different regions which is determined by specific transcriptional programs. This aspect has been largely assessed in previous studies by focusing on the expression of pcGs (Kwan et al., 2012; Bijanzadeh et al., 2018), thus neglecting the potential roles of ncRNAs in both neural development and laminar formation. In order to characterize the ncRNAs expression patterns across different cortical layers, we initially performed cell clustering based on either pcG or ncRNA expression and then merged L2/3 IT, L2/3 IT PPP, and L3 RSP-ACA into layer 2/3 (L2/3), L4/5 IT CTX, L5 IT, L5 NP CTX, and L5 PT CTX into layer 4/5 (L4/5), and L6 CT CTX, L6 IT CTX, L6 IT ENTI, and L6b/CTX into layer 6 (L6) (Figure 3A).

Next, we performed differential expression analysis in L2/3, L4/5 and L6 Glu and observed variations in the proportion of DE gene types across these layers. Notably, the number of DE pcGs and ncRNA were both lowest in L6 (Figure 3B). The proportion of DE pcGs gradually decreased from upper layer to the deeper layers (Figure 3C). Similarly, the proportion of DE lncRNAs also decreased along the layer depth with a sharply decline from 86.8% in L4/5 to 63.2% in L6 (Figure 3C). As expected, these DE pcGs and ncRNAs displayed layer-specific patterns (Figure 3D, Supplementary Figure S3A, and Supplementary Table S3). For instance, the expression of specific genes was determined for layer L2/3 (pcG: *Lpl*; ncRNA: *Gm12371*), L4/5 (pcG: *Trpc3*; ncRNA: *Gm13629*) and L6 (pcG: *Sulf1*; ncRNA: *5330416C01Rik*) (Figure 3E).

To validate the layer-specific genes, we analyzed publicly available ST data obtained from adult mouse sagittal brain slices generated using the Visium platform (See methods). We first performed BayesSpace clustering (See methods) in the whole brain slice (Supplementary Figure S3B) and identified cortical layers based on known markers such as *Calb1* in L2/3, *Rorb* in L4, *Etv1* in L5 and *Tle4* in L6 (Clark et al., 2020; Kozareva et al., 2021; Supplementary Figure S3D). In comparison to non-cortical areas, the cortex demonstrated a higher number of genes (average 5,133) and unique molecular identifier (UMIs) (average 20,617), indicating the high quality of this ST data (Supplementary Figure S3C). A set of layer-specific genes, including pcGs and ncRNAs derived from SMART-Seq v4 data (Figure 3D), were also found to be enriched in their corresponding cortical layers (Figures 3F,G and Supplementary Figures S3E,F), thus confirming the reliability of these layer-specific ncRNAs.

### Cortical region specific ncRNA in mouse cerebral cortex

The cerebral cortex is composed of multiple cortical subregions such as MOp, SSp and VISp, which exhibit distinct functional roles (Hübener, 2003; Li et al., 2015; Rabinovich et al., 2022). The diverse functions of these cortical regions are determined by neural connection, cell composition and gene expression patterns (Nie et al., 2019). While several studies on cerebral cortex have been conducted to study cell composition and function on the basis pcG expression (Jorstad et al., 2023), a systematic exploration of ncRNA expression profiles across cortical regions is currently lacking.

**Figure 4:**
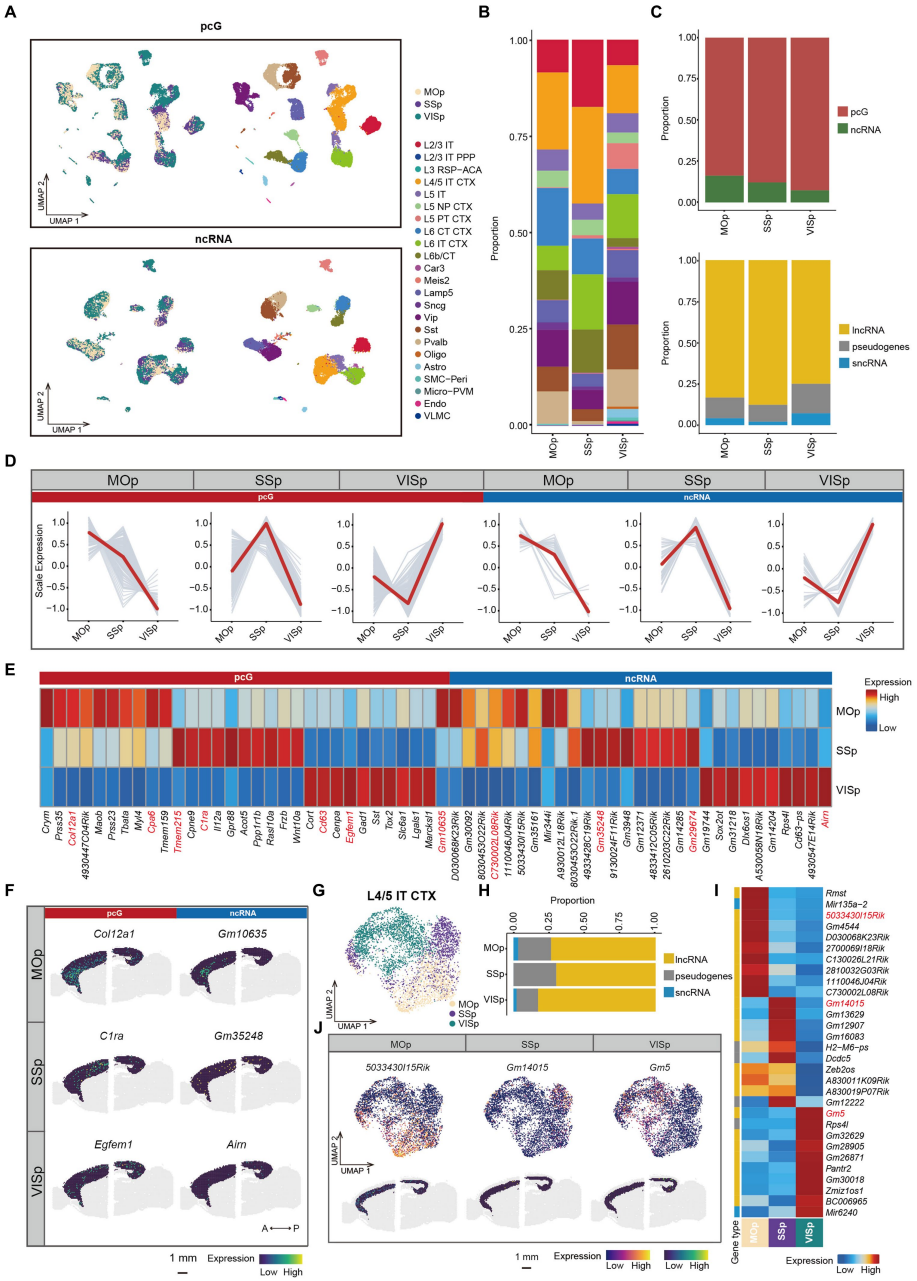
ncRNAs specifically expressed in different cortical areas. **(A)** UMAP visualization of all cells clustered using pcG (top) and ncRNA (bottom), colored by area (left) and cell type (right). **(B)** Stacked bar plots showing the proportion of different cell type in different areas. **(C)** Stacked bar plots showing the proportion of DE gene type in different areas. Top, pcG and ncRNA. Bottom, ncRNA type including lncRNA, sncRNA and pseudogenes. **(D)** Line plot showing area-specifically expressed pcGs and ncRNAs. Gray lines represent the expression dynamics of individual genes and the red line represents the average expression in different areas. **(E)** Heatmap showing the top DE pcGs and ncRNAs of each area. Genes shown in panel **(F)** and Supplementary Figure S4C are marked in red. **(F)** Spatial visualization of the area-specific pcGs and ncRNAs expressed in adult mouse cerebral cortex of 10x Visium ST data. Scale bar, 1 mm. **(G–J)** The ncRNA expression of L4/5 IT CTX is differently expressed in each area. **(G)** UMAP visualization of global clustering of L4/5 IT CTX, colored by area (SSp, MOp and VISp). **(H)** Stacked bar plots showing the proportion of DE ncRNA type in each area. **(I)** Heatmap showing the top DE ncRNAs in each area. **(J)** DE ncRNAs in each area of L4/5 IT CTX. Top, UMAP visualization of ncRNA specifically expressed in MOp, SSp and VISp. Bottom, Spatial visualization of the area -specific ncRNA expressed in adult mouse cerebral cortex of L6. Scale bar, 1 mm.

In this study, we focused on three cortical regions, namely MOp, SSp, and VISp which are located along the anterior–posterior (A-P) axis of the mouse brain (Supplementary Figure S4A). We first performed clustering analysis of the SMART-Seq v4 data obtained from these three regions using either pcG or ncRNA expression (Figure 4A). Next, we calculated the proportion of each cell type in individual cortical region, revealing a significant variation in cell type proportions. Notably, L2/3 IT and L4/5 IT CTX cell types were abundant in the SSp region, while L6 CT CTX was enriched in the MOp. Interestingly, the L5 PT CTX was predominantly observed in the VISp region (Figure 4B). Differential expression analysis conducted among cell types identified specific ncRNAs (*Gm2164*, *9930014A18Rik*, and *Gm26604*) associated with the L5 PT CTX cell type (Supplementary Figure S4B), indicating the possible involvement of those ncRNAs in the formation of region-specific neural circuits. To examine genes that are specific to cortical regions, we merged the SMART-Seq v4 data from cells within the same cortical region to create pseudo-bulk data. By performing differential expression analysis of the cortical regions (Supplementary Table S4), we observed a gradual decrease in the proportion of DE ncRNAs along the A-P axis. However, the overall distribution of ncRNA types across these regions was relatively similar (Figure 4C). Notably, we identified a set of genes that exhibited cortical area specificity not only among pcGs but also among ncRNAs (Figure 4D). Subsequently, these identified genes were also validated using ST data (Figures 4E,F and Supplementary Figure S4C). For instance, in the MOp region, specific genes included *Col12a1* and *Cpa6* (pcGs) as well as *Gm10635* and *C730002L08Rik* (ncRNA), while *C1ra* and *Tmem215* (pcGs) and *Gm35248* and *Gm29674* (ncRNAs) were detected in the SSp region. In the VISp regions, the specific genes consisted of *Egfem1* and *Cd63* (pcG) as well as *Arin* (ncRNA).

Specifically, we observed that cells from same cortical region tended to cluster together in the UMAP, even within the same cell type (Figure 4A). For example, L4/5 IT CTX, L5 IT and L6 IT CTX cells showed a close proximity (Figure 4G and Supplementary Figures S4D,H), indicating that cells of the same cell type acquire substantial differences in terms of transcriptomic programs due to the distinct cortical environment in which they reside. To further investigate the heterogeneity of these neuronal types across different cortical regions, we performed re-clustering and differential expression analysis for each cell type (Supplementary Table S5). For L4/5 IT CTX, we observed a clear separation of cell clusters on the basis of their cortical origin (Figure 4G). The proportion of DE lncRNAs was much higher in the VISp region, while the proportion of pseudogenes was higher in the MOp and SSp regions (Figure 4H). These ncRNAs demonstrated consistent spatial specificity in both single-cell and ST data, exemplified by genes such as *5033430l15Rik* in MOp, *Gm14015* in SSp and *Gm5* in VISp (Figures 4I,J). Similar spatial expression heterogeneity was observed in other cell types, including L5 IT (Supplementary Figures S4D–G) and L6 IT CTX (Supplementary Figures S4H–K). Conversely, there were also cell types that showed minimal or no difference between cortical regions, such as, L5 NP CTX, L6b/CT, Sst, and Lamp5 (Supplementary Figure S4L). In summary, these findings highlight the variations in the coherence of different cell types across cortical regions.

### Functional prediction of ncRNA in the mouse cerebral cortex

ncRNAs exhibit substantial transcriptional activity in the adult mammalian brain and play a crucial role in gene regulation at a broad and complex level (Guennewig and Cooper, 2014). The regulatory mechanisms of ncRNA encompass various processes such as chromatin modification, transcriptional regulation and alternative splicing, among others (Wang and Chang, 2011; Statello et al., 2021). These ncRNA are thought to be the primary driving force behind brain development complexity and cognitive functions (Guennewig and Cooper, 2014; Nie et al., 2019).

**Figure 5:**
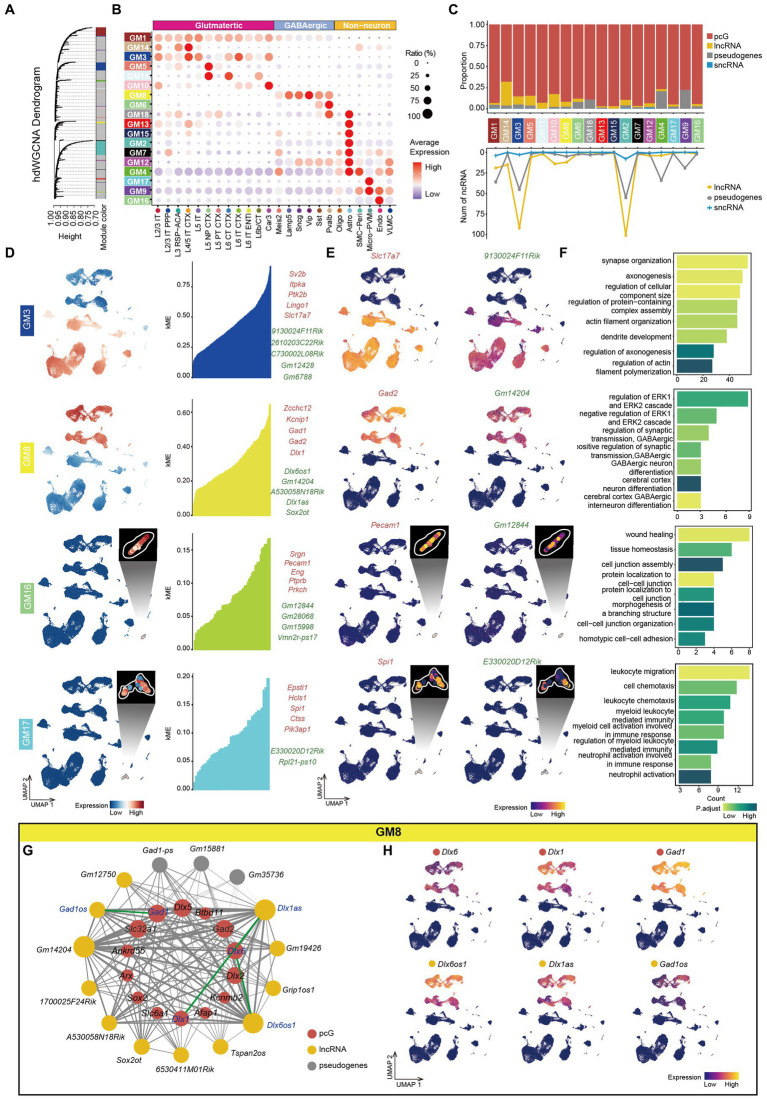
Co-expression networks analysis of mouse cerebral cortex. **(A)** Hierarchical cluster tree showing modules of co-expressed genes identified by hdWGCNA. A total of 18 co-expressed gene modules (GMs) were found and were represented by branches and labeled by different colors to the bottom of the tree. The height (*y*-axis) indicates levels of correlation. **(B)** Bubble plot showing the expression ratio and average expression value of genes in different GMs in each cell type. The color of each bubble indicates the average expression level, and the size indicates the proportion of expressing cells. **(C)** Top, stacked bar plots showing the proportion of gene type in each module. Bottom, line plot showing the number of different types of ncRNA in each module. **(D)** Left, UMAP visualization of average expression level of genes in GM3, GM8, GM16 and GM17. Right: At most top 5 pcGs (red) and ncRNAs (green) in GM3, GM8, GM16 and GM17, ranked by eigengene-based connectivity (kME). **(E)** UMAP visualization of representative pcG and ncRNA in GM3, GM8, GM16 and GM17. **(F)** The bar plot showing the representative gene-ontology (GO) pathways enriched with genes from GM3, GM8, GM16 and GM17, with the color intensity representing the magnitude of the p.adjust value. The *x*-axis represents the number of genes enriched in the pathway, and the *y*-axis represents the name of the enriched pathway. **(G)** The co-expression network showing the interactions between genes in GM8. Nodes represent genes, and edges represent co-expression links. The width of the edges represents the magnitude of the correlation between genes. The size of the nodes represents the number of genes that are mutually associated with that gene. The color of the nodes represents different gene types (pcG: red, lncRNA: yellow, sncRNA: blue, Pseudogenes: gray). The green line represents the connection between sense-antisense gene pairs. **(H)** UMAP visualization of representative pcGs and ncRNAs in GM8.

To gain insights into the function of ncRNAs in the mouse cortex and understand how those ncRNAs coordinate with pcGs to form complex networks, we performed high dimensional weighted gene co-expression network analysis (hdWGCNA) (See methods) using the total RNA data from all cell types. The optimal soft-power threshold was determined at 5, which corresponded to the elbow point of the curve (Supplementary Figure S5A). After filtering, we retained strong connection relationships and identified 18 gene co-expression modules (GMs) (Figure 5A and Supplementary Table S6). Correlative analysis between GMs revealed high correlation among certain modules (Supplementary Figure S5B). We also calculated the gene score of each GM across all cell types (Figure 5B) and observed that many GMs were specifically enriched in particular cell types. For example, GM1 was enriched in neuron, GM3 in Glu, GM8 in GABA, GM9 in Non-neu, GM5 in L5 NP CTX, GM10 in Car3, GM16 in Endo and GM17 in Micro-PVM. Generally, the ratio of ncRNA was consistently lower across all modules (Figure 5C). Among the ncRNAs, Glu-related modules (GM14, GM3, GM5, GM11, and GM10) exhibited a higher proportion of lncRNA compared to pseudogenes whereas Pvalb (GM6 and GM18), Astro (GM4) and Non-neu (GM9) related modules were predominantly composed of pseudogenes (Figure 5C). Moreover, the top 5 pcGs in many GMs ranked by eigengene-based connectivity (kME) were cell type-specific marker genes, such as *Slc17a7* (Glu) in GM3, *Gad1* and *Gad2* (GABA) in GM8, *Etv1* (L5 Glu) in GM5, *Pecam1* (Endo) in GM16 and *Spi1* (Micro-PVM) in GM17 (Figure 5D and Supplementary Figure S5C). Intriguingly, GM9, a module related to Non-neu, was enriched with ribosome- and mitochondria-associated genes but not cell type-specific genes (Supplementary Figure S5C). The expression patterns of the top ncRNAs selected in this manner were highly consistent with their corresponding pcG marker genes. For instance, *9130024F11Rik* from GM3 was specifically enriched in Glu cells, consistent with the expression pattern of *Slc17a7* (Figure 5E). Similar patterns were observed for the top ncRNAs in the GABA-related module (GM8), Endo-related module (GM16) and Micro-PVM-related module (GM17), aligning with the expression pattern of their respective pcG marker genes (Figure 5E).

Furthermore, we performed GO enrichment analysis on the genes within each module and identified corresponding functions that were in line with their associated cell type. For instance, GM3, enriched in Glu cells, was associated with synapse organization, axonogenesis and dendrite development, while GM8, enriched in GABA cells, was related to GABA differentiation and regulation of synaptic transmission (Figure 5F). Similarly, GO function enriched in Non-neu was also consistent with their cell identity. For instance, GM16 (Endo) was correlated with protein localization to cell–cell junction and wound healing and GM17 (Micro-PVM) was correlated with the regulation of myeloid leukocyte-mediated immunity (Figure 5F).

To explore potential connections between ncRNAs and pcGs within individual GM, we constructed a gene interaction network by calculating the strength of co-expression relationships. In GM8, enriched in GABA cells (Figure 5D), the network consisted of 14 pcGs, 12 lncRNAs and 5 pseudogenes (Figure 5G). Notably, 3 ncRNAs (*Gm14202*, *Dlx1as* and *Dlx6os1*) exhibited higher number of connections with genes in the module (Figure 5G). Two of these ncRNAs (*Dlx1as* and *Dlx6os1*) had a counterpart (*Dlx1* and *Dlx6*) in sense-antisense RNA pair. Additionally, another pair of sense-antisense genes (*Gad1os* and *Gad1*) was also included in the network. The strength of connections between each pair of the sense-antisense genes was relatively strong (Figure 5G). All three pairs of genes were exclusively expressed in GABA cells, as expected (Figure 5H). However, the expression patterns of each pair GABA cell subtypes varied (Figure 5H). *Dlx6* was a slightly higher in MGE-derived GABA (Pvalb and Sst GABA), while *Dlx6os1* was significantly higher in non-MGE-derived GABA (Sncg, Lamp5 and Vip GABA). Among subtypes of non-MGE derived GABA, *Dlx1* was more abundant in Vip while *Dlx1as* was relatively higher in Lamp5. For the last pair of the genes, *Gad1* and *Gad1os* demonstrated the highest expression level in Lamp5 and the lowest in Sst, although there was a significant difference in expression levels between the gene pair, which may be due to shared promoter of these two genes.

In summary, we utilized co-expression and GO enrichment analysis to predict the function of ncRNAs. This approach provides a valuable data resource for further understanding the function of ncRNAs in cortical cells.

### The association between ncRNA and neurological disorders

**Figure 6:**
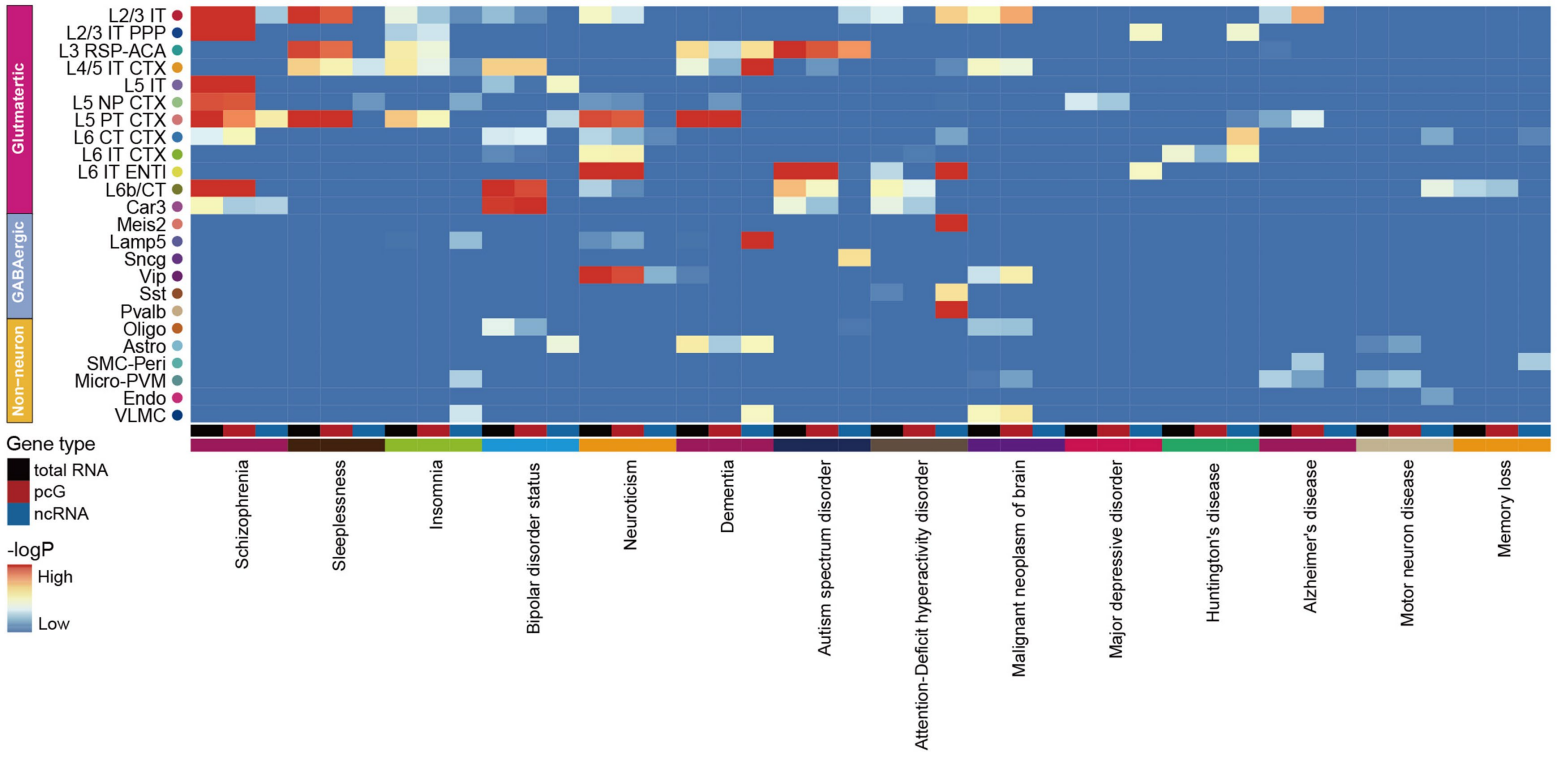
Association of mouse cortex cell transcriptomic profiles with human neurological disorders. The heatmap shows the association of selected human neurological disorders (indicated at the bottom) with the mouse cortex cell types (indicated at the right) annotated in our dataset by total RNA (black), pcG (red) and ncRNA (blue).

Increasing evidence suggests a strong association between neurodegenerative diseases and the dysfunction or mutations of ncRNAs (Nie et al., 2019; Slack and Chinnaiyan, 2019). In order to evaluate the potential impact of genetic variant loci associated with neurological disorders in specific cell types, we applied GWAS by using 14 human brain disease single nucleotide polymorphism (SNP) loci obtained from the UK Biobank^5^ to calculate the enrichment of selected traits in total RNA, pcG and ncRNA with human homologous coordinates (Supplementary Table S7). By comparing the enrichment of SNP loci in each cell type with total RNA, we observed that most neurological disorders were closely associated with Glu, including schizophrenia (SCZ), sleep-associated disorders, bipolar disorder, neuroticism, dementia, ASD, attention-deficient hyperactivity disorder (ADHD), major depressive disease (MDD) and huntington’s disease (HD) owing to linked SNPs of these disorders were enriched in Glu, while Alzheimer’s disease (AD) and motor neuron disease both showed an association with immune cells (Figure 6). This finding suggests that Glu are more vulnerable to SCZ, sleep-associated disorders, bipolar disorder, neuroticism, dementia, ASD, ADHD, MDD and HD compared to Non-neu, whereas immune cell are more susceptible to AD and motor neuron disease, which is consistent with previous reports (Campisi et al., 2022; Han et al., 2022). Notably, we observed a similar enrichment pattern of neurological diseases across cell types comparing total RNA and pcG (Figure 6). However, when analyzing ncRNAs, we identified a distinct enrichment pattern of these diseases in different cell types, and certain disorders were exclusively linked to ncRNAs, such as dementia with Lamp5, ASD with Sncg, ADHD with Sst and Pvalb, and MDD with L2/3 IT PPP and L6 IT ENTI (Figure 6).

Furthermore, we found that the level of enrichment in certain cell types was higher in total RNA and ncRNAs than in pcGs. For example, bipolar disorder showed higher enrichment of SNPs in total RNA and ncRNA within L5 IT. Dementia exhibited this kind of enrichment pattern in L3 RSP-ACA, L4/5 IT CTX and Astro. ADHD and HD displayed this kind of enrichment pattern in L6 IT ENTI and L6 IT CTX, respectively. This finding suggested that neurological disease associated variants that located in ncRNAs may be a co-factor to cause dysfunction of brain cells.

In summary, when investigating the pathogenesis and treatment of neurodegenerative diseases, particular attention should be given to ncRNAs and the specific cell types in which they are specifically expressed. Our study provides additional insights into the role of ncRNAs in pathogenesis of neurological diseases.

## Discussion

A comprehensive understanding of the expression characteristics of ncRNAs is crucial for elucidating their role in maintaining normal brain activity and uncovering the pathogenesis of various neurological disorders. scRNA-seq techniques greatly expanded our knowledge of gene expression at single cell resolution. Existing databases of ncRNA expression patterns in the mouse brain primarily rely on *in situ* hybridization staining from results of Allen brain atlas (Mercer et al., 2008), which has limitations in capturing a wide range of ncRNAs due to probe design strategies. To address this, we conducted a systematic analysis of ncRNA expression patterns at single-cell resolution using SMART-Seq v4 data from 18 mouse cortical regions. We analyzed a total of 11,596 ncRNAs, including 3,865 lncRNAs, 6,706 pseudogenes and 1,025 sncRNAs. It is worth noting that SMART-Seq v4 cannot capture non-polyadenylated RNAs, such as transfer RNA and circular RNA, so our analysis is limited to transcribed ncRNAs. Future advancements in single-cell sequencing technologies are needed to explore the full spectrum of ncRNAs at single cell level.

We identified numerous ncRNAs with cell type, cortical layer and cortical region specificity. Some of those findings were validated using published single cell / singe-nucleus RNA-seq data and 10x visium ST data. We also identified ncRNAs that are specific to cortical regions within the same cell type, such as *5033430l15Rik* in MOp for L4/5 IT CTX, *Gm14015* in SSp and *Gm5* in VISp. Our study expanded the current database of ncRNAs by identifying ncRNAs with high specificity in cell type, cortical layer and cortical region, which expanded the current ncRNA resource database. In our study, ncRNAs specifically expressed in three cortical regions (MOp, SSp and VISp) along the cortical A-P axis have been depicted and more efforts are needed to investigate more brain regions and discover new region-specific ncRNAs.

Given the complexity of ncRNA regulatory mechanisms (Statello et al., 2021), our current understanding of their functions in the cerebral cortex is limited. In our study, we identified a total of 18 GMs through hdWGCNA and then inferred the functions of ncRNA by utilizing pcGs as a bridge. Previous studies had demonstrated that sense–antisense pairs can form intricate reciprocal regulatory circuits to modulate gene expression (Song et al., 2020). Interestingly, we identified three sense-antisense relationships in GM8, among which the *Dlx1* and *Dlx1as* pair was confirmed to involved in the synthesis of GABA, synaptogenesis, and dendritic development of GABAergic neurons (Kraus et al., 2013), supporting the reliability of this approach to predict the potential function of ncRNAs. However, it is indeed necessary to add functional validation experiments via specific transgenic mouse or CRISPR interference for those ncRNA in the future to show clear physiological function of these cell type specific ncRNA.

ncRNAs have been implicated in the development of neurological diseases including ASD, AD and others (Nie et al., 2019; Ma et al., 2020; Ghafouri-Fard et al., 2022). These studies establish a connection between ncRNAs and complex mental diseases. In our study, we integrated GWAS data to identify vulnerable cortical cells associated with multiple neurological disorders by calculating SNP enrichment in total RNA, pcG and ncRNA, respectively. The distinct enrichment pattern of ncRNAs in cortical cells suggests an intricate pathogenesis underlying these diseases. While our analysis was conducted exclusively on mouse data, future investigations should incorporate data from the human cerebral cortex for a more accurate interpretation of the results. The ncRNAs and cell types associated with these diseases may serve as candidates for pre-diagnosis and treatment, offering a new direction for exploring brain diseases that requires further in-depth exploration.

In summary, this atlas of ncRNA expression in the mouse brain provides valuable insights into the role of ncRNAs and serves as a powerful resource for both fundamental and clinical research in the field of ncRNAs.

## Data availability statement

The original contributions presented in the study are included in the article/Supplementary material, further inquiries can be directed to the corresponding authors.

## Ethics statement

The animal study was approved by the Institutional Review Board of BGI. The study was conducted in accordance with the local legislation and institutional requirements.

## Author contributions

YW: Writing – original draft, Writing – review & editing. JL: Writing – original draft, Writing – review & editing. YP: Writing – review & editing. HC: Writing – review & editing. TL: Writing – review & editing. YT: Writing – review & editing. LW: Writing – review & editing. HW: Writing – review & editing. GV: Writing – review & editing. LL: Supervision, Writing – review & editing. LH: Supervision, Writing – review & editing.

## Footnotes

^1^ https://portal.brain-map.org/atlases-and-data/rnaseq/mouse-whole-cortex-and-hippocampus-smart-seq

^2^ https://github.com/bulik/ldsc/wiki/LD-Score-Estimation-Tutorial

^3^ https://genome.ucsc.edu/cgi-bin/hgLiftOver

^4^ https://github.com/bulik/ldsc/wiki/Cell-type-specific-analyses

^5^ https://nealelab.github.io/UKBB_ldsc/downloads.html

## Supplementary material

Supplementary figure s1 Overview of single-cell transcriptome data in mouse cerebral cortex. **(A)** Pie chart showing the proportion of gene type in single-cell transcriptome data in mouse cerebral cortex. **(B)** Boxplot showing the count number of genes for all cells at the total RNA, pcG, and ncRNA level. **(C)** Boxplot showing the count number of genes in each cell type at the total RNA, pcG, and ncRNA level. **(D)** UMAP visualization of all cells clustered using pcG (top, res = 2.0) and ncRNA (bottom, res = 2.0) respectively, colored by cluster. **(E)** Heatmap showing the top DE pcGs (left) and ncRNAs (right) of each cell type. Genes shown in Figure 1E are marked in red.

Supplementary figure s2 Specifically expressed ncRNA in cell types of Glu/GABA/Non-neu. **(A)** Pie chart showing the proportion of class including Glu, GABA and Non-neu. **(B)** Histogram showing the number of differentially expressed (DE) genes per class with pcG (red) and ncRNA (blue). DE genes were defined as genes with log2 (fold change) ≥ 0.25 (light color bars) or > 0.75 (dark color bars) and FDR-adjusted *p*-value < 0.05. **(C)** UMAP visualization of ncRNAs specifically expressed in Glu, GABA and Non-neu. **(D)** UMAP visualization of all Non-neu clustered using ncRNA, colored by cluster (res = 0.5) (left) and UMAP visualization of all cells clustered using pcG, colored by same cluster from left. Cells cycled in left panel were projected to the right UMAP plot. **(E)** UMAP visualization of known markers expressed in Micro and PVM. **(F)** UMAP visualization of ncRNAs specifically expressed in Micro and PVM. **(G)** UMAP visualization of all cluster of adult mouse MOp 10x cell V2 (left) / V3 (right) single-cell data, colored by cell types. **(H)** UMAP visualization of ncRNAs specifically expressed in Glu cell types, GABA cell types, and Non-neu cell types in 10x single cell RNA-seq (scRNA-seq) data. **(I)** UMAP visualization of ncRNA specifically expressed in Glu cell types, GABA cell types, and Non-neu cell types between SMART-Seq v4 (top) and 10x scRNA-seq data (bottom).

Supplementary figure s3 Spatial visualization of layer-specifically expressed genes. **(A)** Heatmap showing the top DE pcGs and ncRNAs of each layer. Genes shown in Figure 3E are marked in red. **(B)** 10x Visium spatial transcriptome (ST) in anterior (left) and posterior (right) mouse brain sections, colored by BayesSpace clusters annotated by anatomical regions. Scale bar, 1 mm. **(C)** Violin plot showing the number of genes (left) and UMIs (right) in different layers. **(D)** Spatial visualization of the known markers used to identify L2/3, L4, L5 and L6 in the section shown in B. Scale bar, 1 mm. **(E)** Bubble plot showing layer-specific expression of pcGs (left) and ncRNAs (right) in different layers of 10x Genomics Visium data. The color of each bubble indicates the average expression level, and the size indicates the proportion of expressing cells. **(F)** Spatial visualization of the layer-specific pcGs and ncRNAs shown in E expressed in adult mouse cerebral cortex of ST. Scale bar, 1 mm.

Supplementary figure s4 Specifically expressed ncRNAs in different cortical areas. **(A)** Schematic diagram of the anatomical structure of the Allen adult mouse brain (left) and cortical brain areas (right), including, MOp, SSp and VISp. **(B)** UMAP visualization of L5 PT CTX-specific ncRNAs. **(C)** Spatial visualization of the area-specific pcGs and ncRNAs expressed in adult mouse cerebral cortex. Scale bar, 1 mm **(D–K)**. The ncRNAs of L5 IT **(D–G)** and L6 IT CTX **(H–K)** is differently expressed in each area. UMAP visualization of ncRNA global clustering, colored by area (SSp, MOp and VISp) **(D, H)**. Stacked bar plots showing the proportion of DE ncRNA type in each area **(E,I)**. Heatmap showing the top DE ncRNA in each are **(F, J)**. Differential genes in each area of L5 IT **(G)** and L6 IT CTX **(K)**. Top, UMAP visualization of ncRNA specifically expressed in MOp and VISp. Bottom, Spatial visualization of the area -specific ncRNA expressed in adult mouse cerebral cortex of L5 **(G)** and L6 **(K)**. Scale bar, 1 mm. **(L)** UMAP visualization of ncRNA global clustering of cell types, including L5 NP CTX, L6b CT, Sst and Lamp5, colored by area (SSp, MOp and VISp).

Supplementary figure s5 Co-expression network analysis by hdWGCNA. **(A)** Determination of soft-thresholding power in the hdWGCNA. Left: The plot shows the scale-free topology fit index (y-axis) for different soft-thresholding powers (β) (x-axis). Right: Analysis of the mean connectivity (degree, y-axis) for various soft-thresholding powers (x-axis). **(B)** Heatmap showing the adjacencies of GM. Red represents high adjacency (positive correlation) and blue represents low adjacency (negative correlation). **(C)** Left, UMAP visualization of average expression level of genes in each module. Right: At most top 5 pcGs (red) and ncRNAs (green) in each module, ranked by kME.

Supplementary table s1 Differentially expressed genes between Glu/GABA/Non-neu in mouse cerebral cortex.

Supplementary table s2 Differentially expressed ncRNAs among cell types of mouse cerebral cortex.

Supplementary table s3 Differentially expressed genes among different layers in mouse cerebral cortex.

Supplementary table s4 Differentially expressed genes among different mouse cortical areas.

Supplementary table s5 Differentially expressed genes among different mouse cortical areas within same cell type.

Supplementary table s6 hdWGCNA gene modules in mouse cerebral cortex.

Supplementary figure s7 Association of GWAS human genetic diseases with mouse cortical cell types.
