# Supplementary figures and images for: Expression characteristics and potential function of non-coding RNA in mouse cortical cells
Source: Front Mol Neurosci. 2024 Apr 10;17:1365978. doi: 10.3389/fnmol.2024.1365978 (PMC11040102; doi:10.3389/fnmol.2024.1365978)

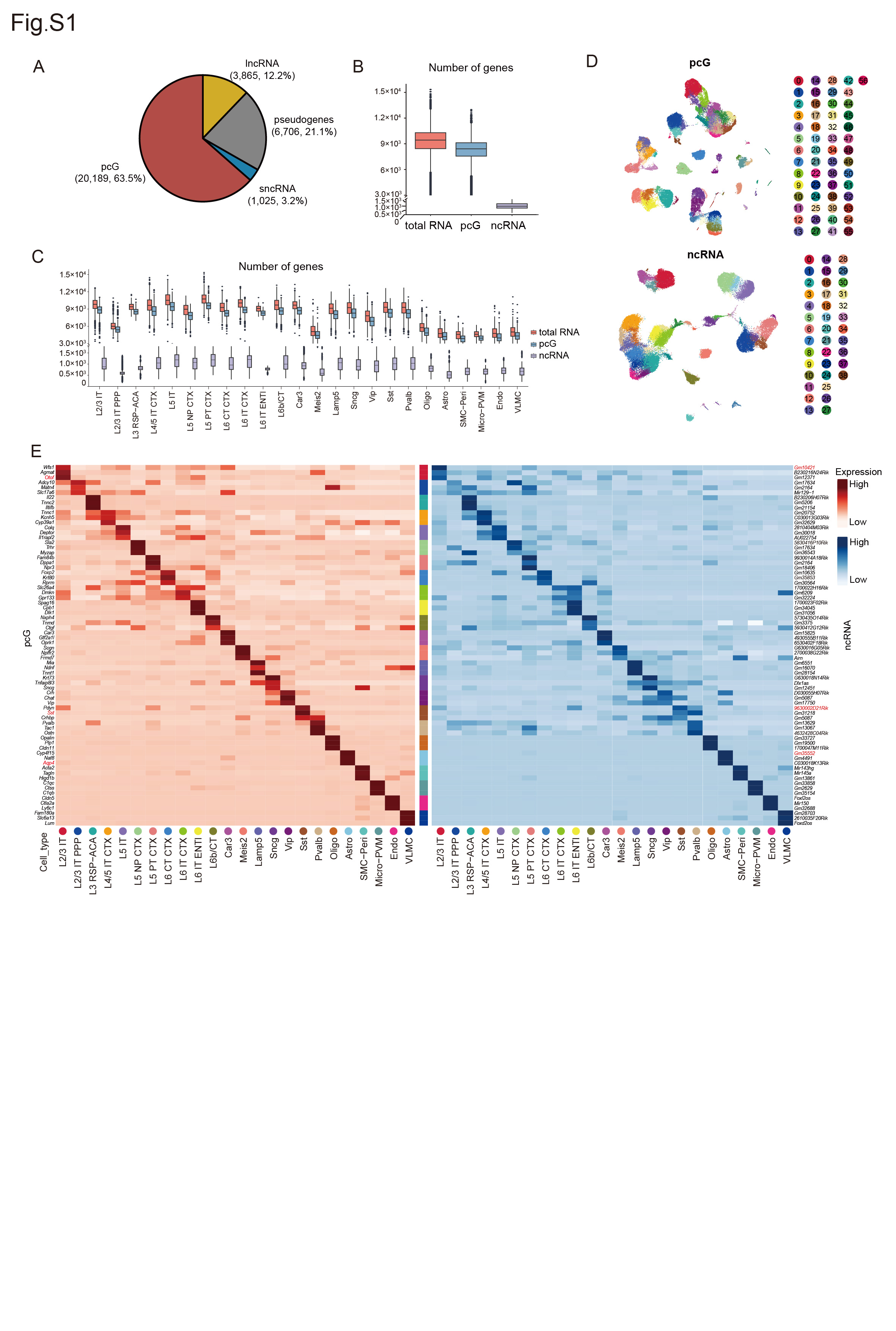

Supplement: Supplementary figure s1 — Overview of single-cell transcriptome data in mouse cerebral cortex. (A) Pie chart showing the proportion of gene type in single-cell transcriptome data in mouse cerebral cortex. (B) Boxplot showing the count number of genes for all cells at the total RNA, pcG, and ncRNA level. (C) Boxplot showing the count number of genes in each cell type at the total RNA, pcG, and ncRNA level. (D) UMAP visualization of all cells clustered using pcG (top, res = 2.0) and ncRNA (bottom, res = 2.0) respectively, colored by cluster. (E) Heatmap showing the top DE pcGs (left) and ncRNAs (right) of each cell type. Genes shown in Figure 1E are marked in red. [file Image_1.JPEG]

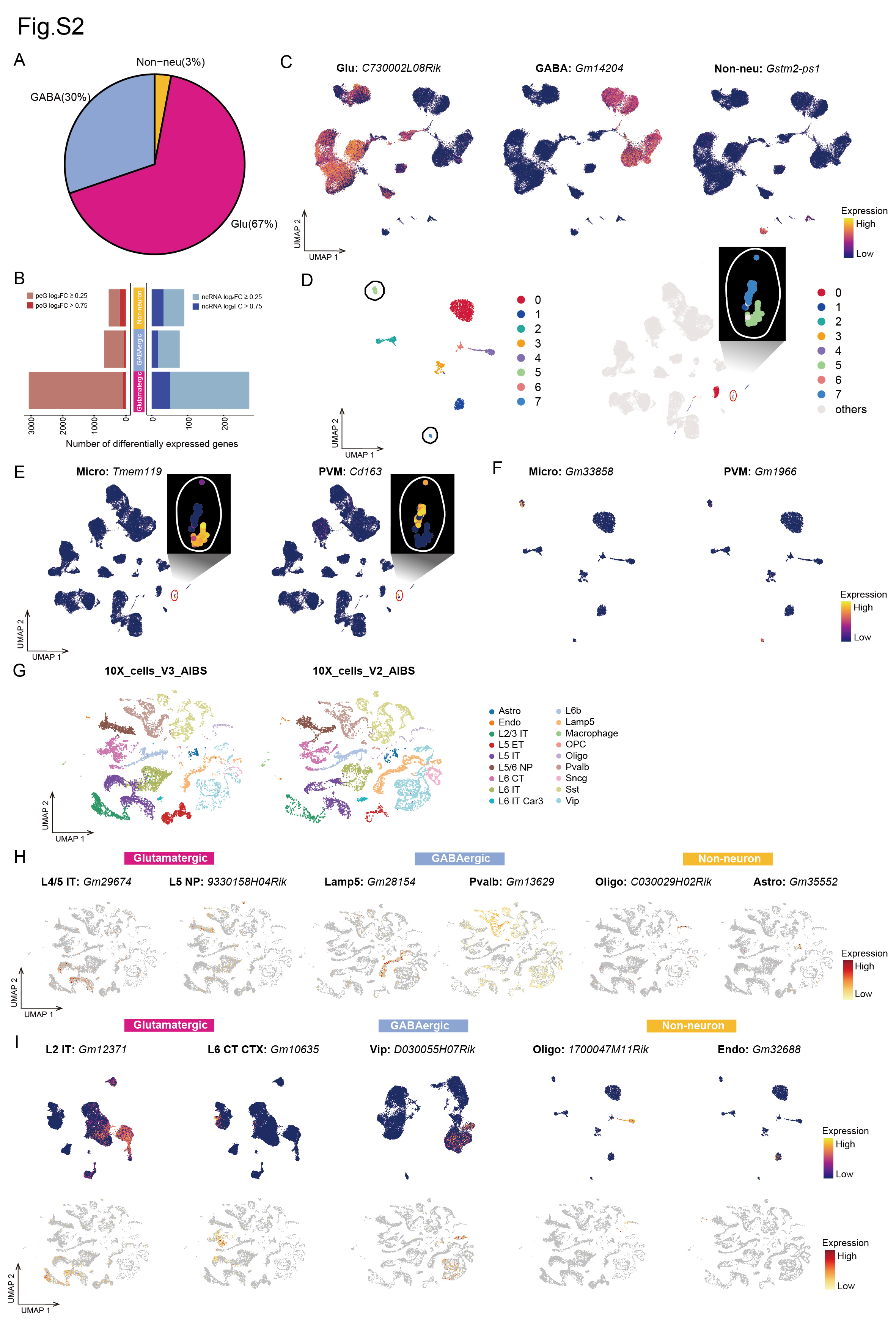

Supplement: Supplementary figure s2 — Specifically expressed ncRNA in cell types of Glu/GABA/Non-neu. (A) Pie chart showing the proportion of class including Glu, GABA and Non-neu. (B) Histogram showing the number of differentially expressed (DE) genes per class with pcG (red) and ncRNA (blue). DE genes were defined as genes with log2 (fold change) ≥ 0.25 (light color bars) or > 0.75 (dark color bars) and FDR-adjusted p-value < 0.05. (C) UMAP visualization of ncRNAs specifically expressed in Glu, GABA and Non-neu. (D) UMAP visualization of all Non-neu clustered using ncRNA, colored by cluster (res = 0.5) (left) and UMAP visualization of all cells clustered using pcG, colored by same cluster from left. Cells cycled in left panel were projected to the right UMAP plot. (E) UMAP visualization of known markers expressed in Micro and PVM. (F) UMAP visualization of ncRNAs specifically expressed in Micro and PVM. (G) UMAP visualization of all cluster of adult mouse MOp 10x cell V2 (left) / V3 (right) single-cell data, colored by cell types. (H) UMAP visualization of ncRNAs specifically expressed in Glu cell types, GABA cell types, and Non-neu cell types in 10x single cell RNA-seq (scRNA-seq) data. (I) UMAP visualization of ncRNA specifically expressed in Glu cell types, GABA cell types, and Non-neu cell types between SMART-Seq v4 (top) and 10x scRNA-seq data (bottom). [file Image_2.JPEG]

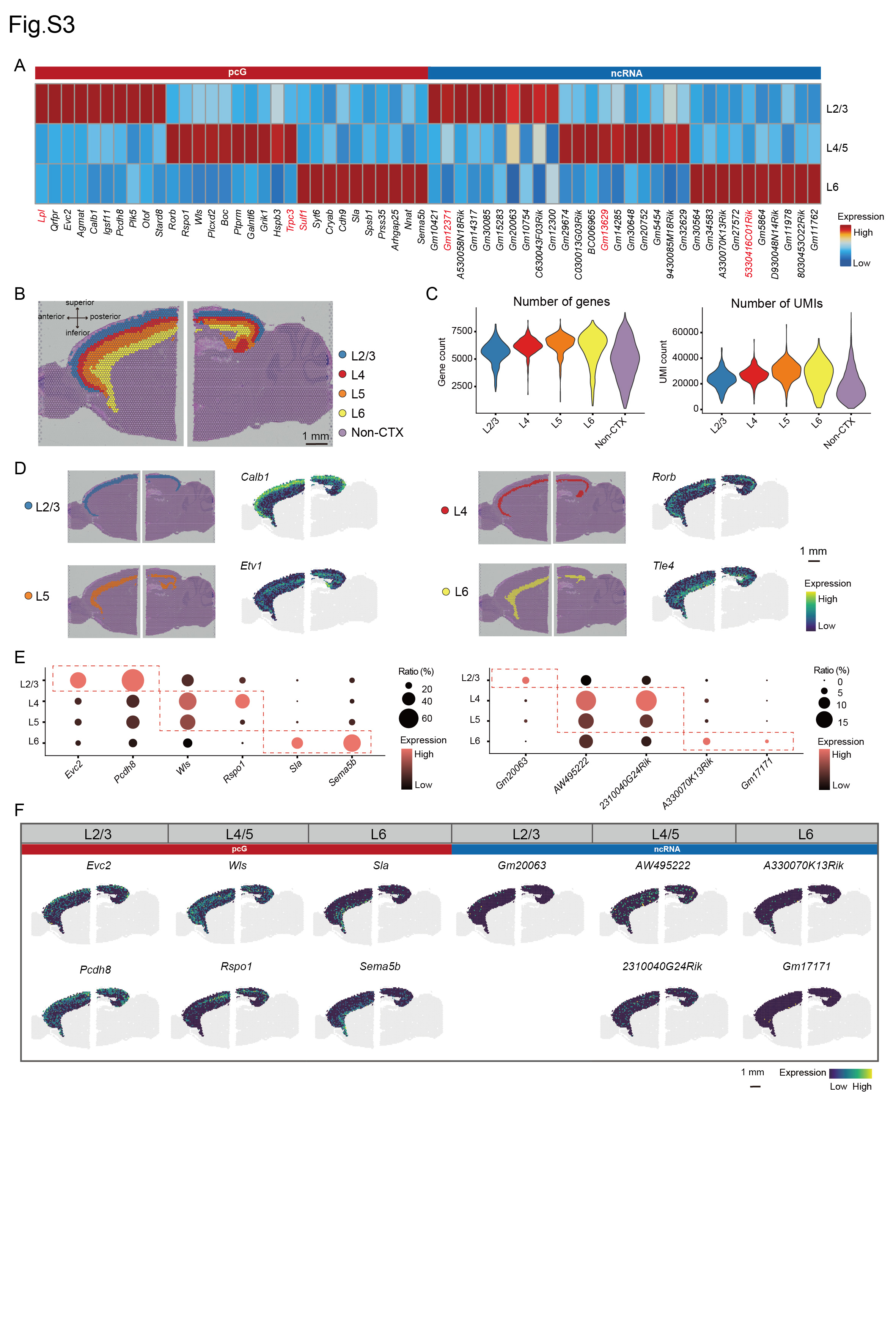

Supplement: Supplementary figure s3 — Spatial visualization of layer-specifically expressed genes. (A) Heatmap showing the top DE pcGs and ncRNAs of each layer. Genes shown in Figure 3E are marked in red. (B) 10x Visium spatial transcriptome (ST) in anterior (left) and posterior (right) mouse brain sections, colored by BayesSpace clusters annotated by anatomical regions. Scale bar, 1 mm. (C) Violin plot showing the number of genes (left) and UMIs (right) in different layers. (D) Spatial visualization of the known markers used to identify L2/3, L4, L5 and L6 in the section shown in B. Scale bar, 1 mm. (E) Bubble plot showing layer-specific expression of pcGs (left) and ncRNAs (right) in different layers of 10x Genomics Visium data. The color of each bubble indicates the average expression level, and the size indicates the proportion of expressing cells. (F) Spatial visualization of the layer-specific pcGs and ncRNAs shown in E expressed in adult mouse cerebral cortex of ST. Scale bar, 1 mm. [file Image_3.JPEG]

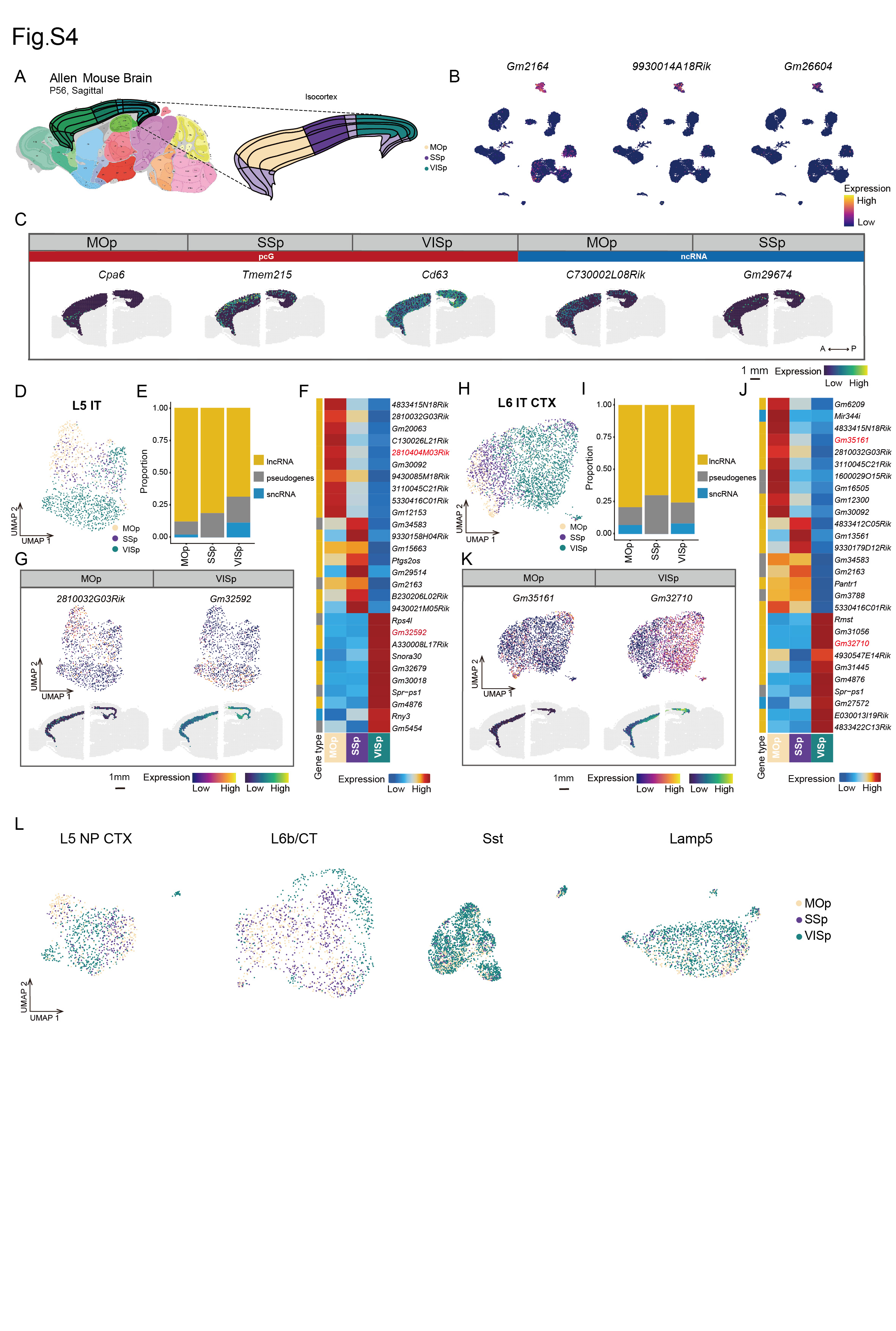

Supplement: Supplementary figure s4 — Specifically expressed ncRNAs in different cortical areas. (A) Schematic diagram of the anatomical structure of the Allen adult mouse brain (left) and cortical brain areas (right), including, MOp, SSp and VISp. (B) UMAP visualization of L5 PT CTX-specific ncRNAs. (C) Spatial visualization of the area-specific pcGs and ncRNAs expressed in adult mouse cerebral cortex. Scale bar, 1 mm (D–K). The ncRNAs of L5 IT (D–G) and L6 IT CTX (H–K) is differently expressed in each area. UMAP visualization of ncRNA global clustering, colored by area (SSp, MOp and VISp) (D, H). Stacked bar plots showing the proportion of DE ncRNA type in each area (E,I). Heatmap showing the top DE ncRNA in each are (F, J). Differential genes in each area of L5 IT (G) and L6 IT CTX (K). Top, UMAP visualization of ncRNA specifically expressed in MOp and VISp. Bottom, Spatial visualization of the area -specific ncRNA expressed in adult mouse cerebral cortex of L5 (G) and L6 (K). Scale bar, 1 mm. (L) UMAP visualization of ncRNA global clustering of cell types, including L5 NP CTX, L6b CT, Sst and Lamp5, colored by area (SSp, MOp and VISp). [file Image_4.JPEG]

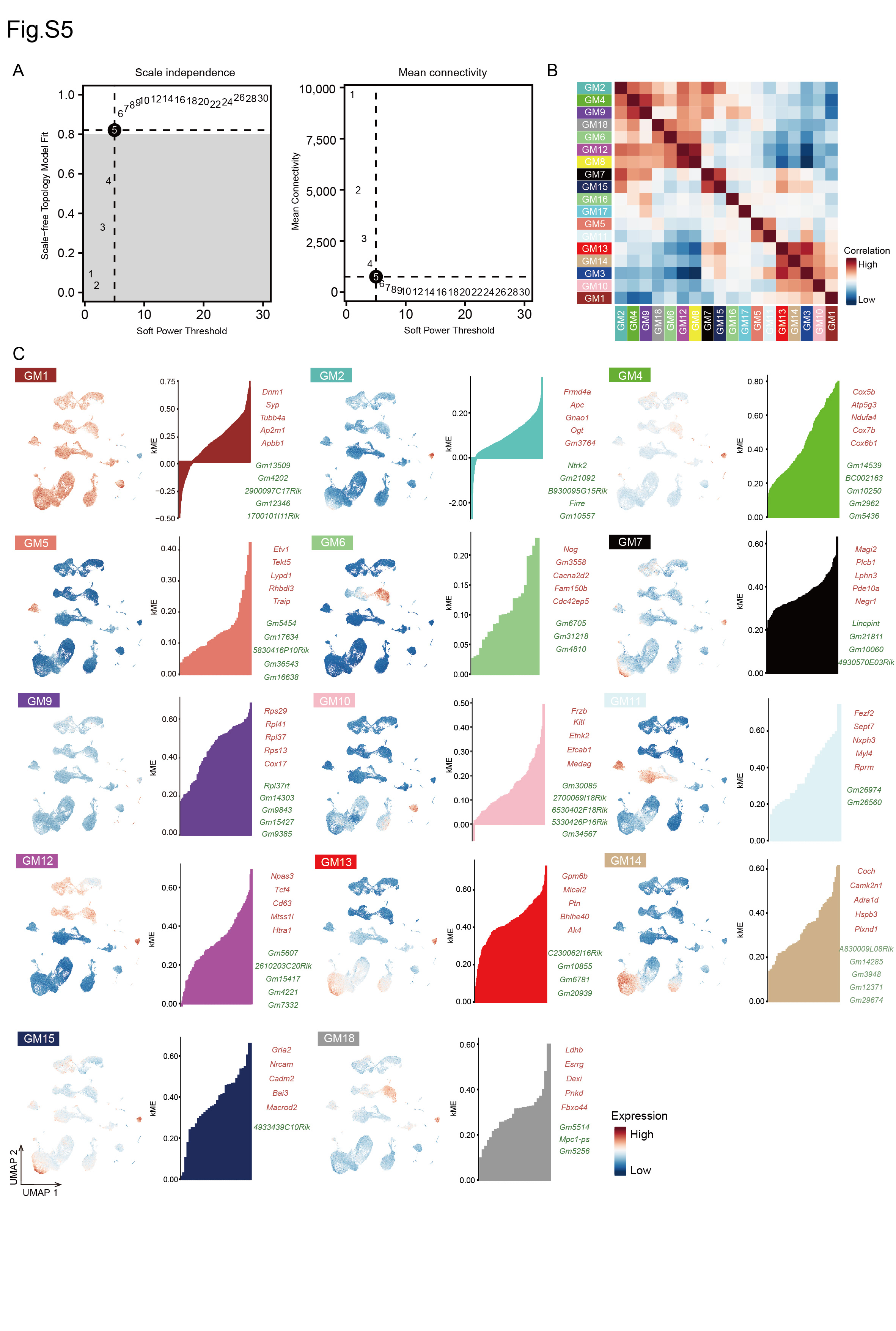

Supplement: Supplementary figure s5 — Co-expression network analysis by hdWGCNA. (A) Determination of soft-thresholding power in the hdWGCNA. Left: The plot shows the scale-free topology fit index (y-axis) for different soft-thresholding powers (β) (x-axis). Right: Analysis of the mean connectivity (degree, y-axis) for various soft-thresholding powers (x-axis). (B) Heatmap showing the adjacencies of GM. Red represents high adjacency (positive correlation) and blue represents low adjacency (negative correlation). (C) Left, UMAP visualization of average expression level of genes in each module. Right: At most top 5 pcGs (red) and ncRNAs (green) in each module, ranked by kME. [file Image_5.JPEG]
